# Enhancement of Adipocyte Browning by Angiotensin II Type 1 Receptor Blockade

**DOI:** 10.1371/journal.pone.0167704

**Published:** 2016-12-19

**Authors:** Kana Tsukuda, Masaki Mogi, Jun Iwanami, Harumi Kanno, Hirotomo Nakaoka, Xiao-Li Wang, Hui-Yu Bai, Bao-Shuai Shan, Masayoshi Kukida, Akinori Higaki, Toshifumi Yamauchi, Li-Juan Min, Masatsugu Horiuchi

**Affiliations:** 1 Department of Molecular Cardiovascular Biology and Pharmacology, Ehime University, Graduate School of Medicine, Tohon, Ehime, Japan; 2 Department of Cardiology, Pulmonology, Hypertension and Nephrology, Ehime University, Graduate School of Medicine, Tohon, Ehime, Japan; 3 Department of Pediatrics, Ehime University, Graduate School of Medicine, Tohon, Ehime, Japan; University of Minnesota Twin Cities, UNITED STATES

## Abstract

Browning of white adipose tissue (WAT) has been highlighted as a new possible therapeutic target for obesity, diabetes and lipid metabolic disorders, because WAT browning could increase energy expenditure and reduce adiposity. The new clusters of adipocytes that emerge with WAT browning have been named ‘beige’ or ‘brite’ adipocytes. Recent reports have indicated that the renin-angiotensin system (RAS) plays a role in various aspects of adipose tissue physiology and dysfunction. The biological effects of angiotensin II, a major component of RAS, are mediated by two receptor subtypes, angiotensin II type 1 receptor (AT_1_R) and type 2 receptor (AT_2_R). However, the functional roles of angiotensin II receptor subtypes in WAT browning have not been defined. Therefore, we examined whether deletion of angiotensin II receptor subtypes (AT_1a_R and AT_2_R) may affect white-to-beige fat conversion *in vivo*. AT_1a_ receptor knockout (AT_1a_KO) mice exhibited increased appearance of multilocular lipid droplets and upregulation of thermogenic gene expression in inguinal white adipose tissue (iWAT) compared to wild-type (WT) mice. AT_2_ receptor-deleted mice did not show miniaturization of lipid droplets or alteration of thermogenic gene expression levels in iWAT. An *in vitro* experiment using adipose tissue-derived stem cells showed that deletion of the AT_1a_ receptor resulted in suppression of adipocyte differentiation, with reduction in expression of thermogenic genes. These results indicate that deletion of the AT_1a_ receptor might have some effects on the process of browning of WAT and that blockade of the AT_1_ receptor could be a therapeutic target for the treatment of metabolic disorders.

## Introduction

It is well known that the renin-angiotensin system (RAS) plays an important role in regulation of hydro-mineral balance and blood pressure control in mammals. Moreover, recent reports have indicated that dysregulation of RAS might be related to the pathogenesis of obesity and adipose tissue dysfunction [1–3]. Angiotensin II (Ang II), a key component in RAS, mediates its action via two subtype receptors, namely the Ang II type 1 receptor (AT_1_R) and the type 2 receptor (AT_2_R). It has been recognized that only rodents have two isoforms of the AT_1_R gene (AT_1a_R and AT_1b_R) [4]. Previous studies revealed that AT_1a_R is predominantly expressed in most tissues [5,6], including adipose tissue, and thus most of the effects of AT_1_R appear to be mediated via the stimulation of AT_1a_R. The importance of Ang II subtype receptors in lipogenesis and lipid metabolism has been demonstrated in recent papers. For example, it has been demonstrated that AT_1_ receptor antagonists facilitated differentiation of 3T3-L1 preadipocytes, with augmentation of glucose uptake [7]. Furthermore, in *in vivo* experiments, blockade of the AT_1_ receptor reduced adipocyte size, with improvement in insulin sensitivity [8], and reduced diet-induced weight gain, and adiposity was attenuated in AT_1a_R knockout mice [9]. We also previously reported that AT_1a_ receptor deletion in atherosclerotic apolipoprotein E knockout mice prevented adipocyte enlargement and enhanced adipocyte differentiation [10]. Conversely, in our previous study, AT_2_ receptor deficiency promoted lipid accumulation and attenuated adipocyte differentiation [11]. Moreover, we demonstrated that AT_2_ receptor stimulation ameliorated insulin resistance, with improvement of adipocyte dysfunction in obese type 2 diabetic mice [12]. These reports indicate important pathophysiological roles of Ang II receptors in adipose tissue, suggesting modulation of these receptors as therapeutic targets for obesity and related metabolic disorders.

Adipose tissue has traditionally been classified into two groups that have directly-opposed capacities; white adipose tissue (WAT) and brown adipose tissue (BAT). WAT is known for its role in the storage of energy in the form of triglycerides, whereas BAT functions for energy expenditure as thermogenesis. In addition, new clusters of adipocytes that express BAT-specific thermogenic genes such as uncoupling protein 1 (UCP1) and have a multilocular lipid droplet appearance have recently been discovered in subcutaneous WAT in response to various stimuli [13,14]. These brown-like adipocytes are designated as ‘beige’ or ‘brite’ adipocytes, and they are similar to classical BAT in that they can burn lipids for energy expenditure. Thus, an increase in beige adipocytes in WAT would increase thermogenic ability and reduce adiposity. Therefore, beige adipocytes have been expected to be a new possible therapeutic target to treat obesity. However, the roles of angiotensin II receptor subtypes in conversion from white to beige adipose tissue have never been explored. To understand the functional roles of Ang II receptor subtypes in browning of WAT, we investigated the effect of genetic ablation of the AT_1a_ receptor or AT_2_ receptor on white-to-beige fat conversion using an animal model.

## Materials and Methods

All procedures were performed in accordance with the National Institutes of Health guidelines for the use of experimental animals. The experimental protocol was reviewed and approved by the Animal Studies Committee of Ehime University.

### Animals and treatment

Adult male C57BL/6J mice (WT), AT_1a_ receptor-deficient (AT_1a_KO) mice (based on C57BL/6J strain and donated by Tanabe Seiyaku Co. Ltd., Japan) and AT_2_ receptor-deficient (AT_2_KO) mice (based on C57BL/6J strain, and provided by Hein et al.) were used in this study [15,16]. In this study, we did not use littermate controls as control. These animals were housed in a room in which lighting was controlled (12 hours on and 12 hours off) and room temperature was kept at 25°C. They were given a standard diet (MF, Oriental Yeast Co., Ltd., Tokyo, Japan) and water ad libitum. At 10 weeks of age, the mice were killed by cervical dislocation, and inguinal white adipose tissue (iWAT) was quickly removed and transferred into liquid nitrogen.

### Morphological analysis of adipose tissue

iWAT was taken and fixed with 10% neutral-buffered formalin solution, and paraffin-embedded sections were prepared [10]. After staining the sections with aldehyde-fuchsin, adipocyte number in five microscopic fields was counted and divided into three groups by cell size (small: 0–200 μm^2^, medium: 200–500 μm^2^, large: >500 μm^2^ using a computer-based imaging system; BZ-II Analyzer (KEYENCE, Osaka, Japan). Values were obtained from six to seven different mice in each group.

### Immunohistochemical staining

Paraffin-embedded cross-sections were immunohistochemically stained using the streptavidin-biotin-peroxidase method. Sections of iWAT were autoclaved (15 min at 120°C) for antigen activation. Endogenous peroxidase and nonspecific binding of the antibody were blocked with 0.3% hydrogen peroxide. The antibody to UCP1 (Abcam, Cambridge, United Kingdom) was applied to the sections, followed by incubation overnight at 4°C. Subsequently, tissue sections were treated with biotinylated secondary antibody (Nichirei, Tokyo, Japan) and then peroxidase-conjugated streptavidin. Positive staining was visualized with diaminobenzidine (Dako Japan, Tokyo, Japan). Counterstaining was performed with hematoxylin.

### Quantitative RT-PCR

Total RNA was isolated from snap-frozen iWAT and differentiated ASC with an RNeasy Lipid Tissue Mini Kit (QIAGEN, Venlo, Netherlands). The extracted total RNA concentration and purity were measured using a NanoPhotometer (Implen, Munich, Germany). cDNA was synthesized from 1 μg of total RNA using a SuperScript VILO cDNA Synthesis Kit (Invitrogen, Carlsbad, CA, USA). Real-time quantitative reverse-transcription polymerase chain reaction (PCR) was performed with a SYBR Primer Ex Taq (Takara Bio Inc., Japan). The sequences of the RT-PCR primers used in this study were as follows: UCP1, 5’-ACTGCCACACCTCCAGTCATT-3’ (forward) and 5’-CTTTGCCTCACTCAGGATTGG-3’ (reverse); Cidea, 5’-TGCTCTTCTGTATCGCCCAGT-3’ (forward) and 5’-GCCGTGTTAAGGAATCTGCTG-3’ (reverse); PGC-1α, 5’-AGCCGTGACCACTGACAACGAG-3’ (forward) and 5’-GCTGCATGGTTCTGAGTGCTAAG-3’ (reverse); PRDM16, 5’-CAGCACGGTGAAGCCATTC-3’ (forward) and 5’-GCGTGCATCCGCTTGTG-3’ (reverse); Tbx1, 5’-GGCAGGCAGACGAATGTTC-3’ (forward) and 5’-TTGTCATCTACGGGCACAAAG-3’ (reverse); Tmem26, 5’-ACCCTGTCATCCCACAGAG-3’ (forward) and 5’-TGTTTGGTGGAGTCCTAAGGTC-3’ (reverse); CD137, 5’-CGTGCAGAACTCCTGTGATAAC-3’ (forward) and 5’-GTCCACCTATGCTGGAGAAGG-3’ (reverse); PPAR-γ, 5’-TGGAGACCGCCCAGGCTTG-3’ (forward) and 5’-GTCTGTCATCTTCTGGAGCACCTT-3’ (reverse); adiponectin, 5’-GCCGCTTATGTGTATCGCTCAG-3’ (forward) and 5’-TGCCAGTGCTGCCGTCAT-3’ (reverse); AT_1_ receptor, 5’-AGTCGCACTCAAGCCTGTCT-3’ (forward) and 5’-ACTGGTCCTTTGGTCGTGAG-3’ (reverse); AT_2_ receptor, 5’-CCTGCATGAGTGTCGATAGGT-3’ (forward) and 5’-CCAGCAGACCACTGAGCATA-3’ (reverse); GAPDH, 5’-TGCGACTTCAACAGCAACTC-3’ (forward) and 5’-ATGTAGGCCATGAGGTCCAC-3’ (reverse).

### Cell culture and differentiation

Adipose tissue-derived stem cells (ASC) were isolated from inguinal subcutaneous WAT. Adipose tissue was minced and washed with phosphate-buffered saline (PBS). The minced cells were trapped on fiber mesh and cultured in ASC growth medium (Mirai Bio Kobo, Tokyo, Japan) at 37°C with 5% CO_2_ for 10 days. After 10 days, isolated ASC were plated at a density of 2.0×10^4^ cells/cm^2^ in 6-well plates with 2.0 mL of plating medium (DMEM + 10% FBS). When the cells reached 100% confluence, differentiation was induced by the addition of adipogenic differentiation medium (adipogenic base medium + adipogenic supplement) (R&D Systems). Every 3–4 days, the differentiation medium was removed and replaced. Differentiation was complete after 10 days, at which time adipogenic-induced cells will showed morphological changes and lipid vacuoles. To quantitate or visualize the effect of treatment on lipid accumulation, we performed Oil Red O staining.

### Oil Red O staining

Lipid droplets were stained with Oil Red O (Sigma-Aldrich) as described previously [17]. After being fixed in 10% formalin for 30 min, the cells were washed twice with PBS and stained with Oil Red O solution (4 mg/ml Oil Red O and 60% (v/v) isopropyl alcohol in distilled water) for 15 min. The cells were then thoroughly washed with distilled water before being photographed under an optical microscope. To quantitate lipid accumulation, the incorporated red dye was eluted with 100% isopropyl alcohol and optical absorbance at 540 nm was measured with a spectrophotometer.

### Statistical analysis

All values in the text and figures are expressed as mean ± S.E. Data were evaluated by ANOVA. If a statistically significant effect was found, post hoc analysis (Bonferroni method or Student's t-test) was performed to detect the difference between the groups. Values of p <0.05 were considered to be statistically significant.

## Results

### Effects of angiotensin receptor subtype deletion on body and adipose weight and inguinal white adipose tissue morphology

**Fig 1:**
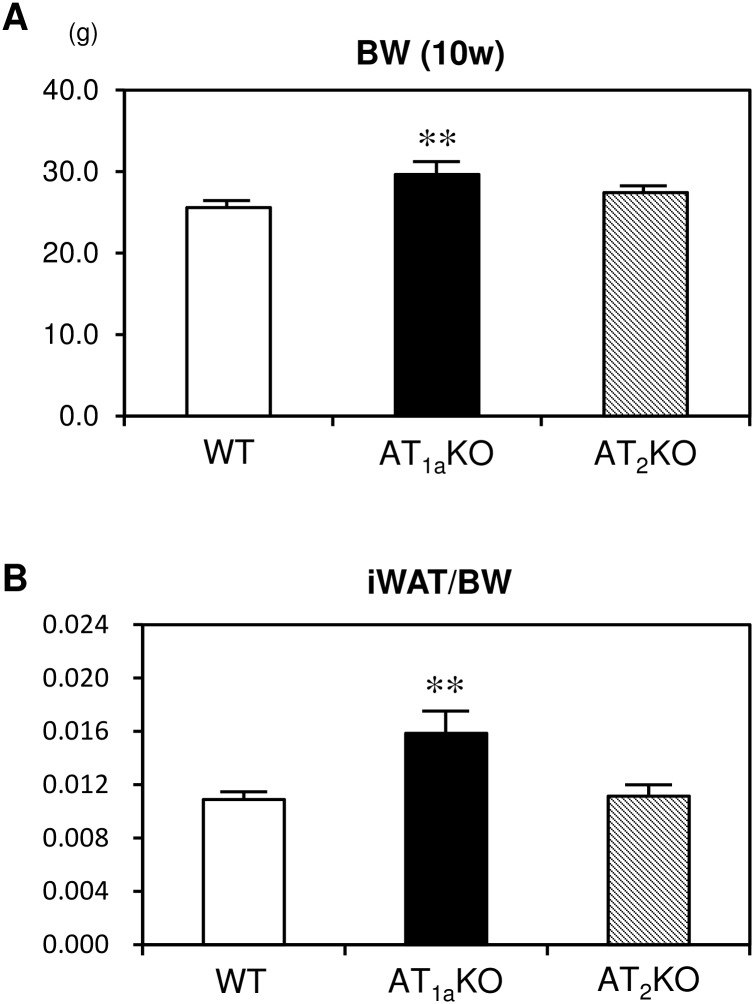
Effect of angiotensin subtype receptor deletion on body weight and adipose tissue weight. (A) Body weight at 10 weeks old in WT, AT_1a_KO and AT_2_KO mice. (B) Ratio of iWAT weight to body weight at 10 weeks old in WT, AT_1a_KO and AT_2_KO mice. Data are expressed as mean±SEM. n = 15 for each group. **P<0.01 vs. WT.

**Fig 2:**
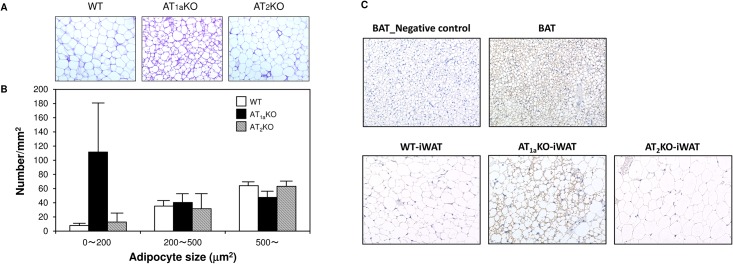
Effect of angiotensin subtype receptor deletion on inguinal white adipose tissue morphology. (A) Representative images of aldehyde-fuchsin staining and UCP1 immunohistochemical staining of iWAT. (B) Adipocyte number in each cell size group (0–200, 200–500, >500 μm^2^). Data are expressed as mean±SEM. n = 5 for each group. (C) Representative images (×400) of UCP1 immunohistochemical staining of brown (BAT) and inguinal white adipose tissue.

First, we examined whether angiotensin receptor subtype deletion affects body and iWAT weight. At 10 weeks of age, body weight and iWAT/body weight ratio were significantly increased in AT_1a_KO mice compared with WT mice (Fig 1A and 1B). On the other hand, there was no marked difference in body weight and iWAT/body weight ratio between AT_2_KO and WT mice. We next investigated the effects of angiotensin receptor subtype deletion on iWAT morphology. Multilocular lipid droplets, characteristic of beige adipose tissue, were more clearly observed in iWAT from AT_1a_KO mice, although AT_1a_KO mice showed a significant increase in iWAT/body weight ratio (Fig 2A). Fig 2B showed that the number of small size cells was higher in AT_1a_KO mice compared with other mouse strains. Moreover, immunohistochemical analysis showed that these small-sized lipid cells were UCP1-positive adipocytes, which indicated that iWAT of AT_1a_KO mice might be converted to beige adipose tissue (Fig 2C). However, these UCP-1 positive adipocytes were observed partially in iWAT, not in whole tissue. In WT and AT_2_KO mice, iWAT was mainly composed of unilocular cells filled with a large droplet, characteristic of white adipocytes. Protein level of UCP1 in iWAT was also investigated by Western blotting. Although UCP1 was abundantly expressed in BAT; however, UCP-1was undetectable in iWAT of all groups (data not shown).

### AT_1a_R deletion increases mRNA expression of white-to-beige fat conversion-specific markers in iWAT

**Fig 3:**
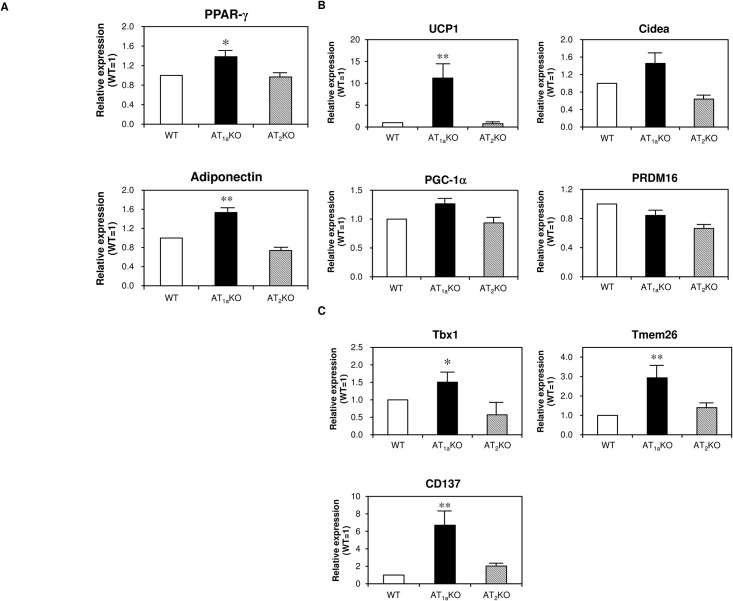
mRNA expression in iWAT of WT, AT_1a_KO, and AT_2_KO mice. (A) Expression of adipocyte differentiation factors in iWAT. (B) Expression of thermogenic genes in iWAT. (C) Expression of beige-selective genes in iWAT. Data are expressed as mean±SEM. n = 15 for each group. *P<0.05, **P<0.01 vs. WT.

We hypothesized that change of iWAT morphology was induced by adipocyte differentiation, we next examined the expression of mRNAs of PPAR-γ, adiponectin, thermogenic genes (UCP1, Cidea, PGC-1α, PRDM16) and beige-selective genes (Tbx1, Tmem26, CD137) in iWAT by quantitative real-time PCR. The mRNA expression of adipocyte differentiation markers, such as PPAR-γ and adiponectin, was significantly higher in AT_1a_KO mice compared with WT mice (Fig 3A). As shown in Fig 3B, AT_1a_KO mice showed a significant increase in UCP1 mRNA expression compared with WT mice. mRNA expression of other thermogenic genes, such as Cidea and PGC-1α, tended to be higher in AT_1a_KO mice than in WT mice, without a statistically significant difference. Beige-selective genes were all expressed at significantly higher levels in AT_1a_KO mice than in WT mice (Fig 3C). On the other hand, we found no significant differences in these mRNA expression levels between AT_2_KO and WT mice.

### Cell culture experiment using adipocyte-derived stem cells

**Fig 4:**
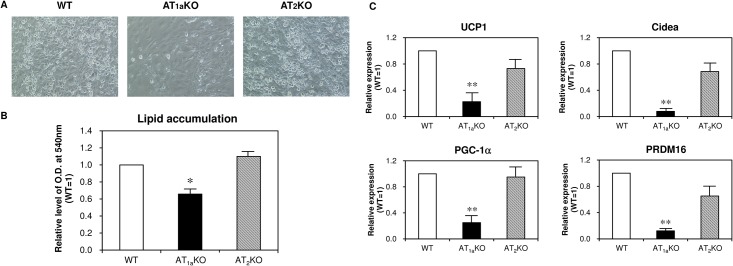
Effect of angiotensin II receptor subtype deletion on adipocyte differentiation from ASC and thermogenic gene expression in differentiated ASC. (A) Representative images of differentiated ASC. (B) Lipid accumulation determined by Oil Red O staining in differentiated ASC. (C) mRNA expression of thermogenic genes in differentiated ASC. Data are expressed as mean±SEM. n = 6 for each group.

To investigate whether angiotensin receptor subtype deletion induces white-to-beige fat conversion at a cellular level, we used adipocyte-derived stem cells (ASCs) isolated from iWAT. As shown in Fig 4A and 4B, ASCs differentiation and lipid accumulation were significantly suppressed in AT_1a_KO mice. Interestingly, mRNA expression levels of thermogenic genes such as UCP1, Cidea, PGC-1α and PRDM16 were decreased in ASC prepared from AT_1a_KO mice compared with those from other mouse strains, which are the distinct results in *in vivo* study (Fig 4C). Deletion of the AT_2_ receptor did not influence these mRNA levels.

### Expression of angiotensin receptor subtypes in white adipose tissue and adipocyte-derived stem cells of WT, AT_1a_ KO and AT_2_ KO

**Fig 5:**
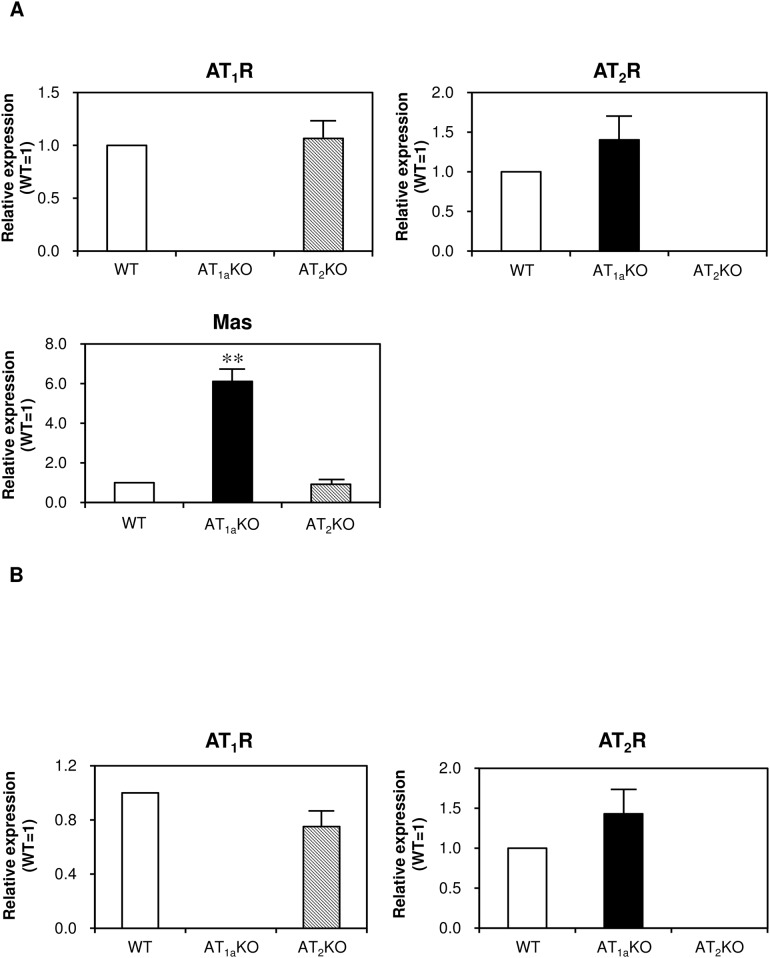
mRNA expression of angiotensin receptors in iWAT (A) and differentiated ASC (B) of WT, AT1aKO, AT2KO mice. Data are expressed as mean±SEM. n = 15 for each group in iWAT. n = 6 for each group in differentiated ASC.

We examined expression of angiotensin receptor subtypes in iWAT (Fig 5A) and ASC-derived adipocytes (Fig 5B). There were no remarkable differences in AT_1a_ and AT_2_ receptor expression between WT and angiotensin receptor knockout mice. mRNA expression of Mas receptor was significantly higher in iWAT of AT_1a_KO mice compared with that in other mouse strains. In contrast, mRNA expression of the Mas receptor was undetectable in ASC-derived adipocytes of any mouse strains (data not shown).

## Discussion

We previously observed in apolipoprotein E-deficient (ApoEKO) mice and KK-Ay mice that blockade or deletion of the AT_1a_ receptor prevents adipocyte enlargement and promotes adipocyte differentiation with the prevention of decrease in adiponectin, PPAR-γ, C/EBPα, and aP2 [10]. We demonstrated in this paper that adipocytes in WAT of AT_1a_KO mice showed an increase in multilocular lipid droplets, with higher expression of thermogenic and beige-selective genes. In contrast, differentiation from ASC into adipocytes was attenuated by AT_1a_ receptor deletion, with decreases in expression of thermogenic and beige-selective genes. These effects mediated by the AT_1a_ receptor were not observed with AT_2_ receptor deletion. These results suggest that AT_1a_ receptor signaling plays an important role in adipose tissue browning.

We observed an increase in body weight and adipose tissue weight in AT_1a_KO mice. de Kloet et al reported that in mice operated by the Cre/lox system to delete AT_1a_R from the paraventricular nucleus of the hypothalamus (PVN), lack of AT_1a_R in the PVN increased food intake and decreased energy expenditure, which augmented body mass and adiposity relative to controls under maintenance on a high-fat diet and reduced hypothalamic inflammation [18]; however, PVN AT_1a_ deletion did not affect body mass or adiposity when the mice were maintained on standard chow. In the present study, we employed conventional AT_1a_ receptor-null mice with standard chow; however, an increase in body weight was observed. AT_1_ receptor signaling is involved in central and peripheral sympathetic nerve activity including in the brain, vasculature, kidney, heart and T-cells [19,20], indicating that deletion of the AT_1a_ receptor in other tissues is intricately associated with body weight and adipose tissue weight. Although we did not investigate catecholamine levels in each mouse tissue, catecholamine levels in urine or serum may reflect the whole sympathetic nerve activity. We assessed the effects of angiotensin receptor subtype deletion on functional difference in adipocytes using O_2_/CO_2_ metabolism-measuring system. There was no significant difference in oxygen consumption between WT mice and AT_1a_KO mice at a normal condition or β_3_-adrenoceptor stimulation (data not shown). In contrast, respiratory quotient was significantly reduced in AT_1a_KO mice compared with WT mice at a normal condition, while respiratory quotient was similar in each mouse after β_3_-adrenoceptor stimulation by CL 316,243 (data not shown). These results indicated that fat oxidation relatively increase in AT_1a_KO; however, basal metabolism did not increase remarkably in AT_1a_KO. Moreover, rectal temperature was significantly lower in AT_1a_KO compared with WT and AT_2_KO mice (data not shown). These results suggest that compensatory response via fat oxidation is occurred to prevent body weight gain due to less sympathetic nerve activity in AT_1a_KO. However, UCP protein levels in iWAT were undetectable. One possible reason is because white-to-beige conversion was only partially induced in iWAT. Another possible reason is because we investigated iWAT from “no-stimulated” mice in the present study. Moreover, distinct results in in vivo and in vitro studies indicate that deletion of AT_1a_ does not affect thermogenic gene expression in a cell autonomous manner, but that the cause of increased thermogenic gene expression may be indirectly mediated through systemic metabolic changes involving sympathetic nerve activity. Further analysis is necessary in the future.

Matsushita et al. previously reported that blockade of endogenous Ang II by an AT_1_ receptor blocker inhibited adipogenesis [21,22]. Moreover, genetic disruption or pharmacological inhibition of the AT_1_ receptor attenuated atherosclerosis and improved endothelial function in diabetic ApoEKO mice via the PPAR-γ pathway [23]. Our previous report demonstrated that AT_1a_ receptor deletion in ApoEKO mice prevented the decrease in expression of PPAR-γ [10]. PPAR-γ is one of the critical transcription factors that initiates a cascade of adipocyte differentiation [24], suggesting that deletion of the AT_1a_ receptor may affect ASC differentiation partly via the PPAR-γ pathway. On the other hand, we observed that expression of PPAR-γ in iWAT was higher in AT_1a_KO mice. PPAR-γ is also a key regulator of white-to-brown adipogenesis [25]. Ang II is associated with activation of NF-κB-mediated genes and downregulation of PPARs [26]. Lack of AT_1a_ receptor-induced inflammatory response may increase PPAR-γ in adipocytes and enhance adipocyte-browning.

We also observed that mRNA expression of the Mas receptor was significantly increased in white adipose tissue of AT_1a_KO mice. Murcas et al reported that Ang 1-7-treated rats given a high-fructose/low-magnesium diet (HFrD) had lower body weight, total fat mass, and serum triglycerides, improved glucose tolerance, and better insulin sensitivity, and that fully developed adipocytes were present in most HFrD myofiber cultures but entirely absent in cultures from Ang 1-7-treated rats [27]. Therefore, an increase in expression of the Ang-(1–7)-Mas axis might also play a possible role in adipocyte differentiation.

In conclusion, we assumed that blockade of the AT_1a_ receptor could have some effects on browning of WAT, with inhibitory effects on ASC differentiation into adipocytes. Therefore, it is possible that blockade of AT_1_R may be useful for the treatment of obesity and metabolic syndrome by enhancing adipocyte browning.
